# MesoScreen: A Computational Platform Providing Unified Data for Liquid-Crystal Research

**DOI:** 10.3390/molecules31203602

**Published:** 2026-10-10

**Authors:** Keda Yang, Longkun Du, Zirui Lv, Wangshi Ning, Jun Li, Qiongyan Shao, Jie Qu, Lihui Hong, Teng Lu, Xiaoling Xu, Denghui Li

**Affiliations:** 1 Key Laboratory of Artificial Organs and Computational Medicine of Zhejiang Province, Shulan International Medical College, Zhejiang Shuren University, Hangzhou 310015, China; 2 State Key Laboratory of Chemistry for NBC Hazards Protection, Beijing 102205, China; 3 Computer Network Information Center, Chinese Academy of Sciences, Beijing 100190, China

**Keywords:** MesoScreen, liquid crystals, molecular dynamics, unified property dataset, liquid-crystal design

## Abstract

The development of advanced liquid-crystal (LC) materials requires reliable and standardized structure–property data, yet generating such data through molecular simulation remains time-consuming and technically demanding. Here, we present MesoScreen, an online computational platform that converts SMILES-encoded molecular structures into standardized simulation workflows. Under consistent conditions, MesoScreen generates unified multi-property profiles including molecular polarizability, orientational order and phase, density, birefringence, and a director mean-square-displacement-based estimator of rotational viscosity. The platform was evaluated on 36 rod-like LC compounds. PCH5 at 313 K serves as a temperature- and phase-matched experimental anchor. Across three chemically distinct molecular cores, lateral substitution consistently reduces orientational order while leaving polarizability anisotropy relatively unchanged, leading to lower calculated birefringence within the Vuks framework. MesoScreen thus provides a scalable and reproducible framework for standardized LC property screening and cumulative dataset construction, supporting virtual screening and future data-driven materials research.

## 1. Introduction

Thermotropic liquid crystals represent a unique class of molecular materials that combine long-range orientational ordering with fluid-like mobility [1]. Their ability to respond to external stimuli, including electric fields, temperature variations, and mechanical perturbations, has enabled widespread applications in display technologies, optical modulation devices, photonic systems, and emerging adaptive materials [2,3,4]. Among various liquid crystal architectures, rod-like calamitic mesogens remain particularly important because their molecular rigidity, anisotropic shape, and electronic conjugation provide direct control over key functional properties such as birefringence, dielectric anisotropy, phase transition temperature, and rotational viscosity [5,6,7,8].

However, the molecular design of high-performance liquid crystals remains challenging because many desirable properties are intrinsically coupled. For example, increasing molecular conjugation and polarizability anisotropy can enhance optical birefringence [6,7], while simultaneously increasing molecular rigidity and intermolecular interactions that may lead to larger rotational viscosity and slower electro-optical switching [8]. Therefore, efficient exploration of the multidimensional structure–property landscape requires computational approaches capable of evaluating multiple physical properties simultaneously [9].

Computational chemistry and atomistic molecular dynamics provide an effective route to generating liquid-crystal property data under controlled conditions [9,10,11,12,13]. In practice, however, the route from a molecular structure to an equilibrated condensed-phase simulation remains labour-intensive. Geometry preparation, charge derivation, force-field assignment, system packing, annealing, production, and property-specific analysis must all be completed before a single comparable record is obtained. These preparatory demands limit the rate at which non-specialist liquid-crystal researchers can construct consistent computational datasets.

Machine learning (ML) and artificial intelligence (AI) are increasingly used for property prediction and inverse design in soft and liquid molecular materials [14,15]. A recent liquid-crystal-focused perspective further identifies AI as a route toward accelerated property prediction, formulation, and experimental decision-making [16]. Such models require more than a collection of published values: they require molecular identities, matched conditions, consistent definitions, provenance, uncertainty, and explicit reliability labels. Heterogeneous data generated at different temperatures, wavelengths, force fields, and phase states cannot be treated as interchangeable training targets.

An online standardized workflow can address both limitations. First, it reduces repeated manual preparation by converting a SMILES-defined molecule into a reproducible simulation and analysis sequence. Second, it creates a common data specification in which direct observables, fitted estimates, phase classifications, and formula-derived quantities remain distinguishable. This distinction is essential because a deterministic transformation, such as Vuks-derived birefringence, must not be validated against the same variables used in its defining equation.

Here, we present MesoScreen as a SMILES-to-record computational platform for five central liquid-crystal property groups: static molecular polarizability; orientational order and phase assignment; density; Vuks-derived birefringence; and a director-MSD-based estimator of rotational viscosity. The present study uses 36 numbered rod-like mesogens, plus three non-mesogenic controls, to evaluate protocol consistency, expose property-specific failure modes, and define a reliability-aware initial seed dataset for cumulative data-driven research. Reliability in this work means traceable inputs, standardized conditions, explicit derivations, and visible applicability limits; it does not imply that every reported value has already achieved experimental accuracy.

The contributions of this work are fourfold.

(i) An online SMILES-to-record workflow that reduces the manual preparation required for quantum-chemical calculations, force-field generation, packing, annealing, production, and analysis.
(ii) Unified reporting of five property groups under predefined conditions, with explicit separation of primary observables, phase classifications, fitted estimates, and deterministic transformations.
(iii) A layered evaluation strategy using orientational order, density, the director-MSD estimator, the internal homologous subset, and selected experimental anchors.
(iv) A standardized, reliability-labelled 36-compound initial seed dataset and machine-actionable record specification, designed for cumulative growth; at its present size it is not intended for training or comparative evaluation of ML models.

## 2. Results and Discussion

### 2.1. System Functionality and Interface

MesoScreen is implemented as a SMILES-to-record web platform. A user submits a molecule as a SMILES string or draws it in the embedded editor; the record is queued with a stable original identifier. The public site comprises a submission page, a queue board that lists pending and completed jobs, and a results table that displays, for each finished molecule, the structure image, SMILES, scalar order parameter *S*, heuristic phase label, an MD snapshot, molecule count, cell volume, density, polarizability anisotropy Δα, HOMO–LUMO gap Δ*E*, Vuks birefringence Δ*n*, and rotational viscosity γ_1_. A detail page shows the same quantities with larger images. The interface is multilingual; submitter e-mail addresses are stored for notification but are not displayed.

**Figure 1:**
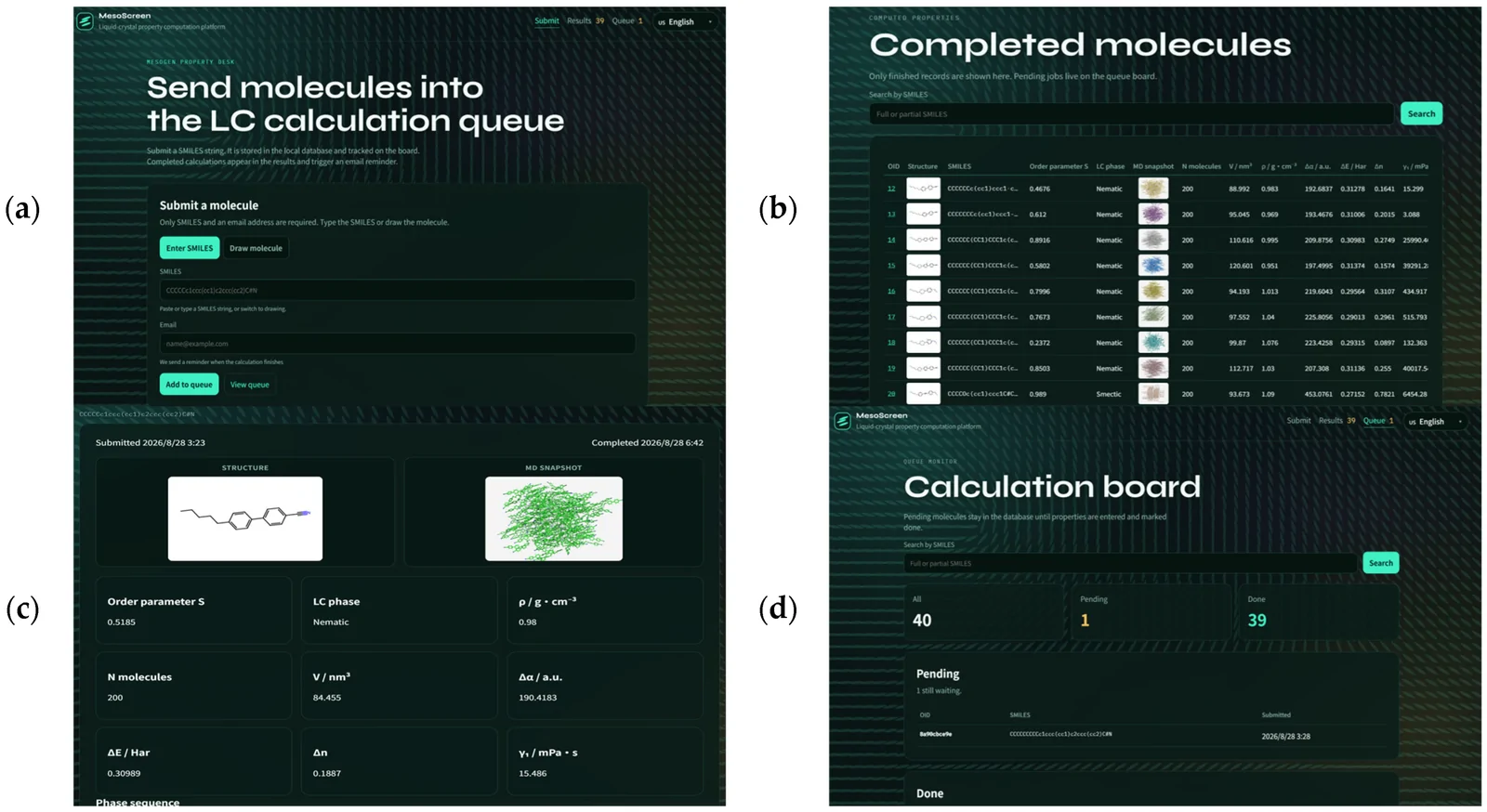
MesoScreen web interface. (**a**) SMILES submission; (**b**) completed-results table; (**c**) 5CB detail page with structure and MD snapshot; (**d**) queue board.

Behind the interface, a compute server applies the Liquid Crystal Predictor classifier of Wu et al. [17] to predict if the input SMILES is liquid crystal. MesoScreen uses it only as a pre-filter, so residual false positives cost computer time rather than data quality, while the low false-negative rate is what determines screening coverage; molecules outside the classifier’s applicability domain, in particular unconventional architectures under-represented in its training set, may be rejected before MD and can be resubmitted with the filter disabled. Non-mesogens are marked complete and returned as “Not LC” without MD. Putative mesogens enter a fixed pipeline: three-dimensional embedding, AM1 geometry optimization and polarizability in Gaussian, GROMACS packing and molecular dynamics, PyMOL (version: 3.1.0) snapshots [18], Q-tensor/τ1/Vuks/director-MSD analysis, and a push of numerical fields and images back to the website. Local records are then marked complete. The five property groups discussed below are therefore generated under one protocol and one public schema, rather than assembled from unrelated scripts. The public interface is shown in Figure 1.

### 2.2. Dataset Coverage and Workflow Consistency

The workflow was evaluated on 36 numbered rod-like entries (compounds **1**–**36**) plus three non-mesogenic controls returned as Not LC. All MD production runs used the same schedule: high-temperature NPT equilibration at 600 K, annealing from 600 K to 313 K, and 100 ns NPT production at 313 K and 1 bar for 200 molecules. AM1 polarizabilities were taken from Gaussian logs matched by oid. These settings enable comparison across cores and tails, with S, density, heuristic screening phase label and Δn evaluated on the final production configuration and γ_1_ fitted on the production trajectory, so that the structural columns are strictly comparable across compounds even though they carry no statistical uncertainty.

Across compounds **1**–**36**, S ranges from 0.052 to 0.989, density from 0.741 to 1.237 g cm^−3^, static Δα from 29.747 to 810.109 a.u., Vuks Δn from 0.0096 to 0.8636, and apparent γ_1_ from 2.0 to 1.42 × 10^5^ mPa s. Heuristic labels at 313 K comprise five isotropic, three weakly nematic, 23 nematic, one nematic/smectic crossover, and four smectic assignments. These quantities form a unified SMILES-linked record, but they are not one evidence class: S and volume are single-configuration summaries, the phase column is a reproducible heuristic using S and τ1, Δα and ΔE are quantum-chemical, Δn is a Vuks transform, and γ_1_ is a sampling-sensitive fit. Across the 36 compounds, the median MD wall-clock time was 6.95 h per compound, with a mean of 8.82 h on a server with L40 GPU.

Recently, Parton-Barr et al. reported a deep-learning “data-to-molecule” pipeline for fluid ferroelectric (polar nematic) materials [19]. In that work, SMILES strings are converted into molecular graphs; graph-neural-network and fingerprint-based classifiers and regressors, trained on a curated dataset of known longitudinally polar mesogens, predict the occurrence of the ferroelectric nematic phase and its transition temperature; a graph variational autoencoder proposes new structures; and retrosynthetic filtering selects candidates, eleven of which were synthesized and characterized and then fed back into the training set. Both approaches start from a SMILES string, but they obtain predictions in fundamentally different ways. The deep-learning pipeline is statistical: it learns a mapping from structure to experimentally measured phase behaviour and is therefore bounded by the size, chemical coverage and consistency of the available experimental data, while inference is essentially instantaneous. MesoScreen is physics-based: every property is computed de novo for the submitted molecule by quantum-chemical calculation and atomistic molecular dynamics under one fixed protocol, so that no training data are required and each output has a direct microscopic interpretation (orientational order, layering, density, polarizability anisotropy), at a much higher computational cost per molecule (Section 2.2). The two approaches are complementary: learned models can prioritize candidates from large virtual libraries, whereas standardized simulations can supply condition-matched descriptors for shortlisted candidates and, as the dataset grows, physically grounded data for training or validating such models.

### 2.3. Result Validation

#### 2.3.1. Multi-Property of PCH5: Compound **11**

Compound **11** is PCH5(SMILES: CCCCCC1CCC(c2ccc(C#N)cc2)CC1). It is the entry in the present set for which the production temperature falls inside the experimental mesophase with a comfortable margin: PCH5 melts near 303 K and clears near 328 K [20,21], so 313 K lies about 15 K below TNI and roughly 10 K above the melting point. The workflow returns S = 0.466, a nematic label, ρ = 0.936 g cm^−3^, Δα = 113.90 a.u., Δn = 0.0943, and γ_1_ = 105.5 mPa·s satisfying the volume-specific displacement gate (N = 200, V = 90.67 nm^3^). Four of these five outputs can be compared with experiment at the same temperature [20]. The phase label is correct. The order parameter is slightly below the value expected at this reduced temperature. The density is of the magnitude reported for cyclohexyl benzonitriles. The birefringence is 0.0943 against a reported value near 0.120 at 589 nm, a deficit of about 21% whose sign is consistent with the known underestimation of polarizability anisotropy by AM1 and with the slightly low S [20]. Because the comparison is made at a single matched temperature and involves an independently measured optical quantity, it is a genuine consistency test of the workflow rather than a restatement of its own inputs, and PCH5 is therefore adopted as the primary internal anchor of this release.

#### 2.3.2. Lateral Substitution Across Three Cores

A second, structurally independent test is available inside the present dataset. Eight compounds form three small sub-series in which a lateral substituent is added to an otherwise unchanged core: compounds **16**–**18** (pentylcyclohexylphenyl isothiocyanate with zero, one and two lateral fluorines), compounds **25** and **26** (a terphenyl with and without two lateral fluorines), and compounds **14**, **15** and **19** (a cyclohexyl-biphenyl carbonitrile with no lateral group, a lateral methyl, and a lateral fluorine). Table 1 collects the outputs. Because the three cores are chemically unrelated, any trend that repeats across all three is a property of the workflow rather than of one molecule.

First, density rises monotonically with fluorination (1.014 → 1.040 → 1.076 g cm^−3^ in the isothiocyanate series; 1.174 → 1.237 g cm^−3^ in the terphenyl pair) and falls when the substituent is a methyl group (0.995 → 0.952 g cm^−3^), which is the expected consequence of the differing volume and polarizability of the two substituents, and consistent with previous observations [22].

Second, Δα is nearly invariant within each core: the spread is below 6% in every sub-series, confirming that a lateral group does not materially change the polarizability anisotropy along the molecular long axis.

Third, the order parameter falls sharply with lateral substitution, and the reduction tracks the steric severity of the substituent—two fluorines suppress order far more than one (0.800 → 0.767 → 0.237), a lateral methyl more than a lateral fluorine (0.580 versus 0.850), and the doubly fluorinated terphenyl falls below the isotropic threshold altogether (0.470 → 0.074).

The consequence for birefringence is the substantive chemical result of this work. Within each core, Δn collapses by factors of three to seven (0.3107 → 0.0897; 0.2038 → 0.0299; 0.2749 → 0.1574) while Δα changes by less than 6%. Lateral substitution therefore lowers birefringence almost entirely by degrading orientational order, not by altering the molecular polarizability anisotropy. The same conclusion is reached independently in three different chemical families under one fixed protocol, which is precisely the kind of comparison that a standardized workflow makes possible and that heterogeneous literature compilations do not. It also identifies S, rather than the quantum-chemical polarizability, as the dominant source of uncertainty in the Vuks estimate: across the whole set the calculated Δn is low by about 20% where S is near its experimental value (PCH5) [21] and high by roughly a factor of two wherever S exceeds 0.85. Condition-matched primary experimental transition data for compounds **18** and **26** were not identified in the literature used here; their phase labels are therefore retained as screening classifications rather than as experimentally validated assignments.

Literature transition temperatures [1,5,23] support isotropic 2CB–4CB at 313 K and a nematic 7CB near its clearing point (≈42–43 °C). They do not support equilibrium nematic 5CB or 6CB at 313 K (clearing points ≈ 35 °C and ≈29 °C). The internal series is therefore a useful consistency test of chain-length trends in *S*, ρ, and Δ*n*, and simultaneously a demonstration that one-temperature cooling trajectories can retain residual order above *T*_NI_. Odd–even effects and crystal/nematic/smectic windows cannot be mapped from a single temperature. Temperature scans, heating–cooling hysteresis, and replicas are required before homologous phase diagrams are claimed.

#### 2.3.3. Other Molecules

The remaining numbered compounds span cyclohexyl, dioxane, ester, pyrimidine, isothiocyanate, fluorinated biphenyl, and tolane cores. Three submitted non-mesogens (cyclopropane oligomer, ethane, cyclobutane) were classified Not LC and were not sent through MD, which is the intended short-circuit of the pipeline.

**Figure 2:**
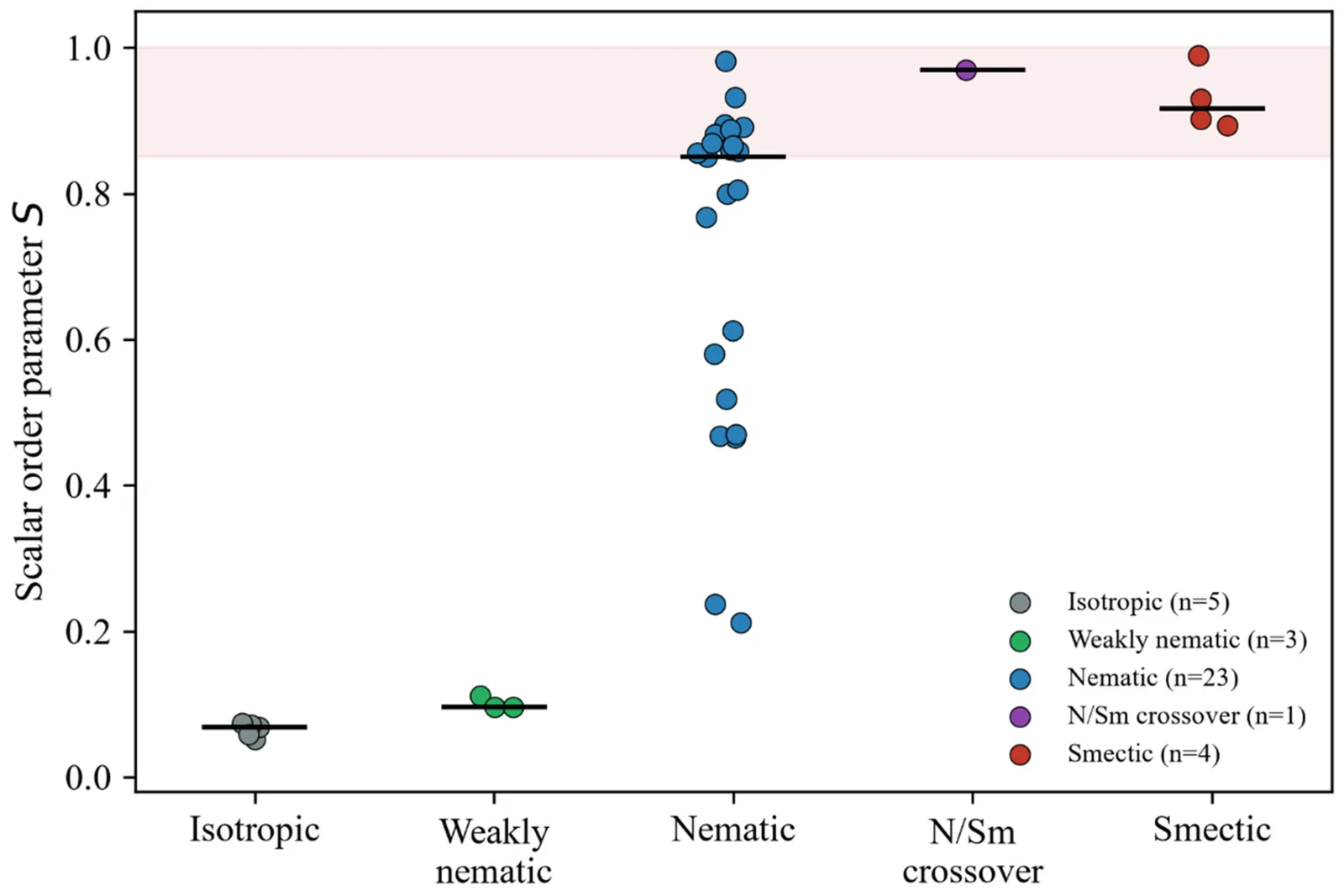
Scalar order parameter grouped by the MesoScreen heuristic phase label for compounds **1**–**36**. Horizontal bars mark group medians. The shaded band (S ≥ 0.85) shows high-order overlap between nematic and smectic assignments.

Among MD-treated structures, low-S isotropic or weakly nematic labels cluster on shorter or laterally bulky molecules (compounds **3**, **4**, **6**, **26** and **28**), whereas extended conjugated rods (compounds **20**, **21**, **31**, **33** and **34**) combine large Δα, high S, and large Vuks Δn. Four smectic labels (**20**, **21**, **27** and **36**) coincide with τ1 ≥ 0.50 rather than with S alone: compound **31** remains labelled nematic at S = 0.982, which is possible only if translational order stays below the smectic threshold (Figure 2). Compound **33** is the single nematic/smectic-crossover assignment. This two-dimensional classifier is a screening result, not a thermodynamic phase diagram. Broad accuracy claims for the full 36-compound set remain premature without a per-compound, condition-matched experimental table; PCH5 (Section 2.3.1) is the matched-state anchor and the lateral-substitution sub-series of Section 2.3.2 supplies the structure–property trend; compounds lying close to their clearing point are treated in Section 4.

## 3. Materials and Methods

### 3.1. Overview of the MesoScreen Automated Workflow

**Figure 3:**
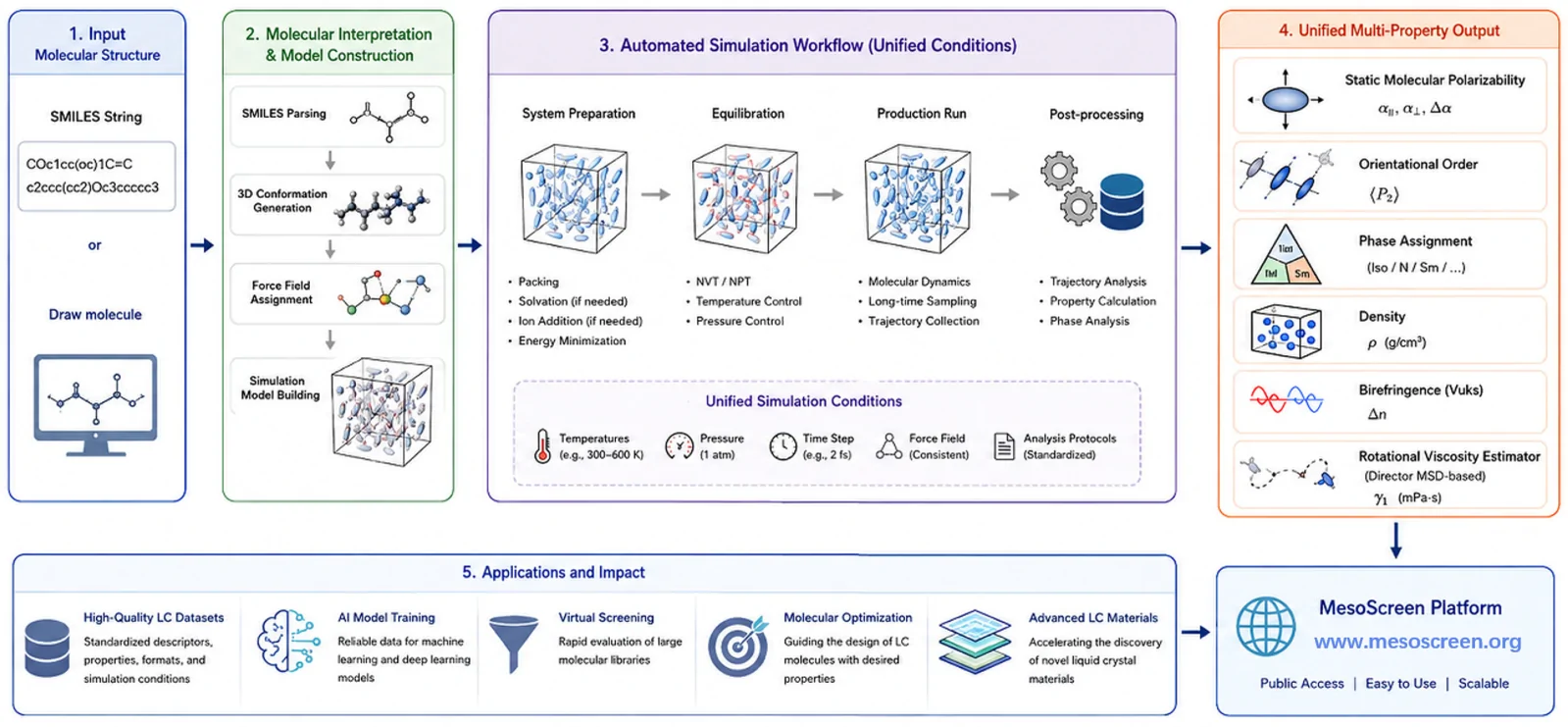
Unified architecture of the MesoScreen computational platform. SMILES-linked molecular identity is propagated through quantum-chemical preparation and standardized MD into five property groups: polarizability, orientational order and phase, density, birefringence, and rotational viscosity.

MesoScreen is an online SMILES-to-record computational platform that links molecular identity to quantum-chemical calculations, force-field generation, molecular dynamics, and property extraction. The five property groups are polarizability, orientational order and phase, density, birefringence, and rotational viscosity. Figure 3 shows the unified architecture of the platform.

Starting from a molecular structure provided as a SMILES string or through the molecular drawing interface, the platform automatically performs molecular interpretation, three-dimensional conformation generation, force-field assignment, and simulation model construction. Molecular dynamics simulations are subsequently executed through a standardized workflow comprising system preparation, equilibration, production simulation, and post-processing under unified simulation and analysis conditions. The resulting trajectories and molecular information are used to generate a unified multi-property profile, including static molecular polarizability, orientational order, phase assignment, density, Vuks-derived birefringence, and a director mean-square-displacement-based estimator of rotational viscosity. By integrating these steps into a single automated pipeline, MesoScreen enables scalable generation of standardized LC data for cumulative dataset construction and virtual screening; once sufficiently large and chemically diverse, such data may also support the development of future ML models.

Protocol consistency follows from the default production settings: the same system size, thermodynamic schedule, non-bonded cutoffs, and production length were used for all compounds. The public website does not expose these parameters to the submitter; they are fixed on the compute server so that records remain comparable.

### 3.2. Molecular Structure Preparation

Submitted SMILES strings were syntax-checked on the web platform. Three-dimensional coordinates were then generated with Open Babel [24] (version: 3.1.0) using the –gen3d procedure, followed by MMFF94 minimization (500 steps). These Cartesian coordinates were the initial geometries for subsequent AM1 optimization and condensed-phase simulation.

### 3.3. Quantum-Chemical Calculations and Molecular Properties

Quantum-chemical calculations were performed by Gaussian09 [25] using the semi-empirical Austin Model 1 (AM1) method [26]. For each molecule, the Cartesian coordinates generated in the preceding molecular-structure preparation step were used to construct an individual Gaussian input file. The calculations were organized as a two-step linked workflow using a common checkpoint file, allowing the optimized molecular geometry and corresponding electronic information to be reused in subsequent property calculations. Unless otherwise specified, the molecules were treated as neutral closed-shell species with a total charge of 0 and a spin multiplicity of 1. Each Gaussian job was configured with 4 GB of memory and four processor cores.

In the first step, the molecular geometry was optimized at the AM1 level using the opt keyword. Molecular symmetry constraints were disabled using nosymm to avoid enforcing symmetry during structural relaxation. The optimized geometry was stored in the Gaussian checkpoint file and subsequently used as the reference structure for all molecular-property calculations.

In the second step, molecular polarizability and dipole-moment properties were evaluated at the same AM1 level using the polar keyword. The optimized molecular geometry was retrieved directly from the checkpoint file using geom = check, while the previously converged wavefunction was used as the initial guess through guess = read. This procedure ensured that the calculated molecular properties were obtained consistently from the optimized structure without reconstructing or independently reoptimizing the molecular geometry. The resulting Gaussian output was used to extract the molecular polarizability (*α*), polarizability anisotropy (Δ*α*), and dipole moment (*μ*).

The molecular polarizability tensor was further characterized in terms of its directional components. For the rod-like mesogens considered in this study, the component along the assigned molecular long axis was denoted as *α*_∥_, whereas the mean polarizability perpendicular to this axis was denoted as *α*_⊥_. The polarizability anisotropy was defined as:

(1) $$\alpha_{∥}=\alpha_{\mathrm{zz}},\alpha_{⊥}=\frac{\alpha_{\mathrm{xx}}+\alpha_{\mathrm{yy}}}{2},\;\Delta \alpha =\alpha_{∥}-\alpha_{⊥}.$$

Here, *α*_∥_ describes the response of the molecular electron distribution along the molecular long axis, *α*_⊥_ represents the average response in the two perpendicular directions, and Δ*α* quantifies the anisotropy of the molecular polarizability. These descriptors were retained as molecular-level properties for subsequent analysis of liquid-crystal optical behaviour. Polarizabilities are reported in atomic units throughout; conversion to the units used in the optical literature follows 1 a.u. = 0.148185 Å^3^.

### 3.4. Automated Force-Field Generation

Molecular-mechanics parameters were generated with the General Amber Force Field 2 (GAFF2) [27]. GAFF2 atom types and AM1-BCC atomic partial charges were assigned with antechamber (AmberTools [28], version 24.8), missing parameters were supplied by parmchk2, and AMBER parameters were converted to GROMACS format with ACPYPE [29] (version: 2023.10.27). GAFF2 was selected for broad chemical coverage and compatibility with scripted parameter generation. Liquid-crystal-specific force fields such as GAFF-LCFF can improve transition-temperature predictions for selected mesogens [30,31], but they cover a narrower chemical space and may require targeted parameterization.

### 3.5. Bulk System Construction

For each compound, 200 molecular copies were packed by PACKMOL [32] into a 15 nm cubic box at low density with a 2.0 Å tolerance, and periodic boundary conditions were applied in all three directions. The initial low-density configurations were energy-minimized by steepest descent with a widened 1.2 nm cutoff, chosen to tolerate residual close contacts from random packing. Identical system size and preparation protocol were applied to every compound.

### 3.6. Molecular Dynamics Simulation Protocol

Molecular dynamics simulations were performed with a GPU-accelerated GROMACS 2024-plumed_2.10.0 [33] using the five-stage protocol in Table 2.

The compression stage increased the low-density packing toward a condensed state. The 600 K stage was intended to erase memory of the initial packing before cooling. Temperature was controlled with the velocity-rescaling thermostat [34] (τT = 0.5–1.0 ps) and pressure with the stochastic cell-rescaling barostat [35] (τP = 2.0–5.0 ps; isothermal compressibility 4.5 × 10^−5^ bar^−1^). Electrostatics used particle-mesh Ewald [36] with a 1.0 nm real-space cutoff; van der Waals interactions used a 1.0 nm cutoff with analytical long-range dispersion corrections; and bonds to hydrogen were constrained with LINCS [37].

Cooling followed a piecewise-linear schedule with nodes at 600, 560, 520, 460, 420, 370, 340, and 313 K at 0, 10, 20, 40, 60, 75, 90, and 100 ns, respectively, corresponding to a mean cooling rate of 2.87 K ns^−1^. Coordinates were written every 10 ps.

### 3.7. Structural Property Analysis

Trajectory analysis was performed with MDAnalysis [38] (version: 2.10.0). In the MesoScreen production pipeline the GROMACS topology (emd.tpr) supplies masses and bonding, and the final production configuration (emd.gro), taken after 100 ns of NPT production at the target temperature, supplies the coordinates for the Q-tensor, translational-order, density and phase-label calculations. Rotational viscosity is evaluated separately from the production trajectory (emd.xtc or equivalent). Evaluating the structural descriptors on the final configuration rather than on a block-averaged window is a deliberate throughput choice for a screening platform: it keeps the per-compound analysis cost negligible relative to the simulation itself, at the cost of not attaching a statistical uncertainty to those descriptors. The same analysis code accepts a multi-frame trajectory, in which case frame-wise values are averaged and a five-block uncertainty is returned; this mode is the basis of the planned block-averaged release. All structural quantities reported here should therefore be read as single-configuration screening descriptors.

A rigid core was identified automatically from the covalent bonding graph. Ring atoms were defined as those incident on non-bridge edges (Tarjan bridge finding) and flexible tails were excluded. If bonding information was unavailable, the whole molecule was used. Atomic masses were taken from the topology rather than inferred from atom names. When bonds were present, coordinates were unwrapped by residue with MDAnalysis before the long-axis calculation, so that molecules split across periodic boundaries did not yield spurious orientations. The molecular long axis was defined as the principal axis of least inertia of the selected core, obtained by diagonalizing the moment-of-inertia tensor. The resulting unit vectors *u*_i_ entered the Saupe Q-tensor analysis described below.

#### 3.7.1. Orientational Order Parameter

The nematic order parameter was obtained from the second-rank ordering tensor built from the per-molecule unit long axes *u*_i_, as defined by the equation:

*Qαβ* = ⟨(3 *uα uβ* − *δαβ*)/2⟩ (2)

The largest eigenvalue of Q is S and its eigenvector is the instantaneous director n; equivalently, S = ⟨(3 cos^2^θ − 1)/2⟩. The difference in the two lesser eigenvalues of Q was recorded as a biaxiality diagnostic. When a multi-frame trajectory is analyzed, frame-wise S values are averaged and the uncertainty is estimated by five-block averaging; an isotropic Monte Carlo baseline (2000 random orientations of the same number of molecules) supplies 95% and 99% apparent-S thresholds against which the measured S is compared. For rotational viscosity, the sign of n is continued between frames to remove the n ≡ −n ambiguity.

#### 3.7.2. Positional Ordering and Phase-Label Provenance

Nematic and smectic phases cannot be distinguished by orientational order alone. A one-dimensional translational order parameter was therefore computed from molecular centres of mass projected on the director, *z*_j_ = *r*_j_·*n*,

*τ*_1_(*d*) = |⟨*exp*(2*πi z_j_*/*d*)⟩| (3)

and maximized over candidate layer spacings *d* from 1.0 nm to min(*L*_box_)/1.5 (400 trial values), thereby excluding sub-nanometre packing noise. Phase labels were assigned heuristically from the mean *S*, the isotropic 95%/99% baselines, and *τ*_1_: isotropic if *S* ≤ *S*_95_; weakly nematic if *S*_95_ < *S* ≤ *S*_99_; nematic, nematic/smectic crossover, or smectic according to whether *τ*_1_ was below 0.30, between 0.30 and 0.50, or at least 0.50. A biaxiality above 0.15 appended a tentative biaxial flag. These labels are screening classifications, not a full thermodynamic phase identification. Each compound record stores the label together with *S*, the number of molecules, the cell volume, and density in the summary CSV.

### 3.8. Density Calculation

Equilibrium density was obtained from the topology masses and the instantaneous simulation-cell volume of the analyzed configuration(s),

*ρ* = *N M*/(*NA* ⟨*V*⟩) (4)

where N is the number of mesogen molecules in the cell (200 in the standard MesoScreen box), M is the molar mass obtained by summing atomic masses from the GROMACS topology, NA is Avogadro’s number, and ⟨V⟩ is the mean cell volume of the analyzed frames (the single emd.gro box in the default batch). Volumes were converted from Å^3^ to nm^3^ before evaluating ρ in g cm^−3^.

### 3.9. Birefringence Estimation

Optical birefringence was estimated from the static molecular polarizability tensor, taken from the corresponding Gaussian log (default match *_AM1.log), and from the MD-derived *S* and cell volume using the Vuks local-field model [39]. Polarizability components were converted from bohr^3^ to Å^3^. The tensor was diagonalized; the largest eigenvalue defined α∥ and the mean of the two lesser eigenvalues defined α⊥, giving Δα = α∥ − α⊥. Directional molecular polarizabilities were then

*αe* = *ᾱ* + (2/3) Δ*α S*,  * αo* = *ᾱ* − (1/3) Δ*α S*,  *ᾱ* = (*α*∥ + 2 *α*⊥)/3 (5)

and the Vuks equations were solved self-consistently for the principal refractive indices,

(*n_i*^2^ − 1)/(⟨*n*^2^⟩ + 2) = 4*π N α_i*/(3 *V*),  ⟨*n*^2^⟩ = (*ne*^2^ + 2 *no*^2^)/3 (6)

with *N* the number of molecules in the cell and *V* the cell volume, giving Δ*n* = *n*_e_ − *n*_o_. Subtracting the two forms of Equation (6) and using α_e_ − α_o_ = Δα·*S* gives

Δ*n* = (4π *N*/3)(Δ*α S*/*V*)(⟨*n*^2^⟩ + 2)/(*ne* + *no*) (7)

Because N is fixed and the refractive-index factor varies slowly, Δn is approximately proportional to ΔαS/V. The calculated Δn is therefore a model-derived transformation of quantities already present in the workflow, not an independent trajectory observable. The HOMO–LUMO gap ΔE was parsed from the same Gaussian log when orbital eigenvalues were present.

### 3.10. Rotational Viscosity Calculation

Rotational viscosity was estimated from fluctuations of the global director, following the statistical-mechanical use of molecular dynamics to estimate nematic rotational viscosity [40]. Per-frame directors were obtained from mass-weighted molecular principal axes after fragment unwrapping; the sign of *n* was continued between frames. For a freely diffusing uniform director mode, the small-angle relation is

⟨|*n*(*t*) − *n*(0)|^2^⟩ = (4 *k_B T*/*γ*_1_
*V*) *t* ≡ *m t* (8)

from which the rotational viscosity follows as

*γ*_1_ *=* 4 *k_B T*/(*V m*) (9)

Here kB is the Boltzmann constant, T is the analysis temperature (default 313 K), V is the cell volume of the first trajectory frame, and m is the fitted MSD slope. The director MSD was evaluated over all time origins by fast Fourier transform, using MSD = 2 − 2·ACF for a unit director. The production defaults are a sampling stride of 25 frames, an MSD window of 20 ns, and a linear fit from 0.5 to 15.0 ns. The slope is rejected if it is non-positive; warnings are issued if the MSD exceeds 0.5 (departure from the small-angle regime) or if the mean S along the viscosity trajectory falls below 0.3. The summary table stores γ_1_ in mPa·s together with the outcome of the volume-specific displacement gate (ΔMSD > 0.02 over the fit window). Records that fail the gate are labelled sampling-limited and are reported as censored lower-confidence outputs rather than as viscosities; the fit R^2^ and the MSD trace are computed internally and are not part of the present public record. Throughout this work γ_1_ is treated as a diagnostic of the workflow rather than as a validated material property. A viscosity estimate remains a descriptive workflow output unless the fit is shown to be linear, diffusive, and within the small-angle regime.

### 3.11. Standardized Record Specification for Cumulative Dataset Construction

For future releases, the atomic unit of the dataset is defined as one molecule–protocol–condition–replicate record. The current MesoScreen record already links a stable original identifier (*oid*) and SMILES string to derived properties *S*, heuristic screening phase label, *N*, *V*, ρ, Δα, Δ*E*, Δ*n*, and γ_1_. Subsequent releases should additionally store the input geometry, protocol version, thermodynamic conditions, replicate identifier, analysis windows, uncertainty estimates, reliability states, and provenance hashes.

Targets should be typed as direct observables, fitted estimates, classifications, or deterministic transformations. This distinction will be essential if the records are later used for ML modelling: Vuks Δ*n* must retain its derivation from Δα, *S*, *V*, and *N*; heuristic screening phase label must remain heuristic classifications unless τ_1_ and the decision rule are exported with the record; snapshot *S* and ρ should not be treated as trajectory-averaged observables; and sampling-limited viscosity values should be represented as censored observations with reliability metadata rather than ordinary continuous targets.

## 4. Applicability Domain and Limitations

**Figure 4:**
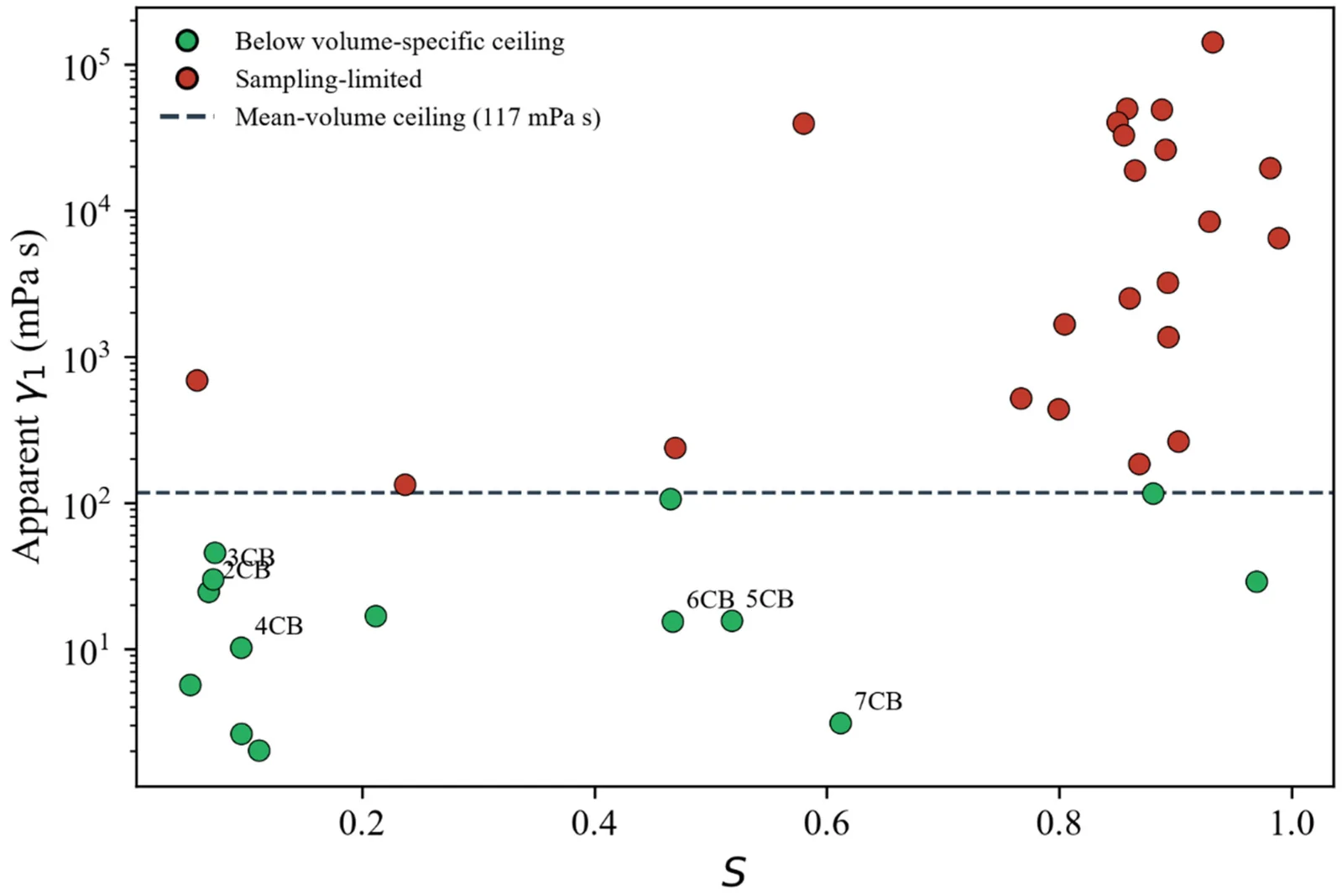
Apparent rotational viscosity versus S. Green: below the volume-specific displacement ceiling; red: sampling-limited. nCB homologues are labelled. 7CB lies numerically below the ceiling but is not treated as validated.

Sampling ceiling for rotational viscosity. The production fit interval is 0.5–15 ns. At the mean cell volume (107.0 nm^3^), requiring ΔMSD > 0.02 over 14.5 ns implies a mean-volume ceiling of approximately 117 mPa s. Apparent viscosities of 10^3^–10^5^ mPa s remain sampling-limited (Figure 4). The nCB values of approximately 10–30 mPa s, except 7CB, fall below the displacement ceiling but still lack exported MSD diagnostics. The parameter γ1 for isotropic snapshots is a director-fluctuation output, not a nematic viscosity.

Proximity to the phase transition. The single-temperature protocol resolves a phase label only when the production temperature is well separated from the equilibrium clearing point. Two entries in the present set are not: 5CB and 6CB clear near 308 K and 302 K, respectively, within a few kelvin of the 313 K production temperature, and both are returned as nematic (S = 0.519 and 0.468) where equilibrium data place them in the isotropic phase [1,5,21]. The remaining cyanobiphenyls behave as expected—2CB and 3CB are isotropic, and 7CB, which clears near 316 K, is nematic—and Δα increases smoothly along the series (183–193 a.u.), so the failure is confined to the transition neighbourhood rather than being a property of the chemical family. Two causes are identifiable: the anneal from 600 K to 313 K is many orders of magnitude faster than any experimental scan, so residual order can survive above TNI; and a label decided within a few kelvin of TNI is set by the force-field error in the clearing point rather than by the workflow. The practical consequence is a stated applicability window rather than a loss of function: outputs are interpreted as mesophase descriptors only where the production temperature lies more than roughly 10 k from the experimental transition, which is the regime of the PCH5 anchor and of all three lateral-substitution sub-series in Section 2.3. Within that window the density, Δα and order-parameter trends reported above are unaffected, and a temperature scan rather than a single-temperature run is the appropriate extension for compounds that sit close to their clearing point. In addition, without systematic sensitivity tests, these phase labels should be regarded as a preliminary screening tool rather than a quantitative thermodynamic assignment.

Four limits define the applicability domain of the present release. First, every structural descriptor except γ_1_ is evaluated on the final production configuration rather than on a block-averaged window, so S, τ_1_, ρ and Δn carry no statistical uncertainty and cannot resolve slow drift; block-averaged records computed from the same trajectories are the first planned extension of the resource. Second, the anneal from 600 K to 313 K is many orders of magnitude faster than any experimental scan, so a high order parameter can reflect kinetic arrest rather than a stable mesophase; the residual order of 5CB and 6CB above their literature T_NI is the clearest illustration, seventeen compounds have S ≥ 0.85 at 313 K and should be read in this light, and temperature scans rather than single-temperature runs are required before phase windows are claimed. Third, GAFF2 was selected for chemical coverage rather than for transition-temperature accuracy, and LC-optimized parameter sets such as GAFF-LCFF [30,31] are expected to reduce this bias; N = 200 is a conventional but unconverged system size, particularly for the layer-resolved smectic assignments. Fourth, birefringence rests on static AM1 polarizabilities and a deterministic Vuks transformation, so Δn inherits the accuracy of Δα, S and V and cannot be validated against those same inputs [6,7]. Accordingly, the present dataset is a standardized, quality-labelled seed intended to grow, not a stand-alone learning benchmark, and none of the five outputs is offered as an experimentally validated prediction.

## 5. Conclusions

MesoScreen is an online SMILES-to-record platform that reduces the preparation required to generate comparable liquid-crystal calculations. Under a shared 313 K protocol it organizes five property groups—polarizability, Q-tensor order and τ_1_-informed phase labels, density, Vuks birefringence, and director-MSD rotational viscosity—together with molecular identity and MD snapshot images.

Evaluation of 36 rod-like mesogens shows why reliability is property-specific. *S* describes alignment but separates nematic from smectic states only when combined with τ_1_. Density is a condensed-phase consistency check. Vuks Δ*n* is a deterministic transform of Δα, *S*, and *V*; a high correlation with those inputs is algebraically expected. Director-MSD viscosities are quantitative only inside a demonstrably diffusive window.

PCH5 supplies the internal matched-state anchor of this release: at 313 K, about 15 K below its experimental clearing point, the workflow returns the correct nematic label, ρ = 0.936 g cm^−3^ and Δn = 0.0943 against a reported 0.120 at 589 nm, a deficit whose sign follows from the static AM1 polarizability. Across three chemically unrelated cores, lateral substitution leaves Δα unchanged to within 6% while reducing S and Δn by factors of three to seven, showing that lateral groups lower birefringence by degrading orientational order rather than molecular polarizability anisotropy, and identifying S as the dominant source of uncertainty in the Vuks estimate. Compounds whose clearing points lie within a few kelvin of the production temperature, notably 5CB and 6CB, fall outside the applicability window defined in Section 4; this bounds the protocol rather than the workflow, and does not affect the trends established inside the window. Non-mesogens are excluded before MD.

The dataset is a standardized, reliability-labelled seed and a specification for cumulative growth, not a completed AI benchmark. By exposing derivation class and failure modes before values enter a training set, MesoScreen can serve as a validation instrument for future data-driven design of liquid-crystalline materials.

## Figures and Tables

**Table 1:** Lateral-substitution sub-series at 313 K. Within each core, Δα varies by less than 6% while S and Δn fall sharply, so the loss of birefringence originates in orientational order rather than in molecular polarizability anisotropy.

No.	Core and Lateral Substitution	ρ (g cm^−3^)	Δα (a.u.)	S	Heuristic Screening Phase Label	Δn
**16**	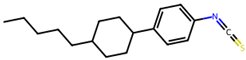	1.014	219.60	0.800	Nematic	0.3107
**17**	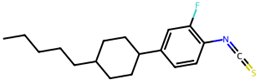	1.040	225.81	0.767	Nematic	0.2961
**18**	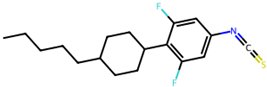	1.076	223.43	0.237	Nematic	0.0897
**25**	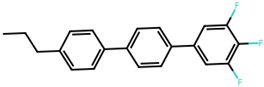	1.174	239.57	0.470	Nematic	0.2038
**26**	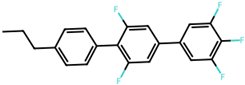	1.237	231.41	0.074	Isotropic	0.0299
**14**	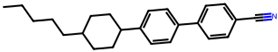	0.995	209.88	0.892	Nematic	0.2749
**15**	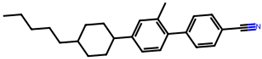	0.952	197.50	0.580	Nematic	0.1574
**19**	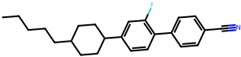	1.030	207.31	0.850	Nematic	0.2550

**Table 2:** Fixed simulation protocol applied to every compound.

Stage	Ensemble	T (K)	P (bar)	Δt (fs)	Length
Energy minimisation	—	—	—	—	steepest descent
Compression	NPT	600	1000	0.5	1 ns
Isotropisation	NPT	600	1	2	10 ns
Annealing	NPT	600 → 313	1	2	100 ns
Equilibrium MD	NPT	313	1	2	100 ns

## Data Availability

The numerical data presented in this study are reported in Table 1 and Table 2 and in Supplementary Tables S1 and S2; numbered molecular structures are provided in Figure S1; representative configurations and the original numerical summary are included in Supplementary Table S1; and the number-to-SMILES mapping is included in Supplementary Table S2. The MesoScreen platform, including the per-compound result pages and images, is accessible without registration at www.mesoscreen.org.

## Supplementary Materials

The following supporting information can be downloaded at: https://www.mdpi.com/article/10.3390/molecules31203602/s1, Figure S1: Numbered two-dimensional molecular structures for compounds **1**–**36**. Table S1: MesoScreen summary values for compounds **1**–**36** at 313 K and 1 bar (200 molecules per system). Compound numbers match website oid values (compound **1** = 5CB). Phase is the S plus τ1 heuristic label. Rotational-viscosity entries are apparent 0.5–15 ns fit outputs; [SL] denotes sampling-limited under the volume-specific ΔMSD > 0.02 check; [DG] denotes that the displacement gate is passed but other diagnostics remain unavailable; † marks the 7CB fit as internally inconsistent with its high S. γ1 for isotropic snapshots is not a nematic viscosity.; Table S2: Standardized molecular identity and mean-density reconstruction for compounds **1**–**36**. Canonical SMILES and standard InChIKeys were generated from the submitted SMILES strings with RDKit. M is the SMILES-derived molar mass. Mean density was reconstructed as ρ = 200 M/(NA⟨V⟩) from the Table S1 cell volume; no density uncertainty is implied because volume time series were not supplied.
