# Supplementary material for: MesoScreen: A Computational Platform Providing Unified Data for Liquid-Crystal Research
Source: Molecules. 2026 Oct 10;31(20):3602. doi: 10.3390/molecules31203602 (PMC13707784; doi:10.3390/molecules31203602)
Supplement: Supplementary file 1 [file molecules-31-03602-s001.zip › molecules-4577957-supplementary.pdf]

MesoScreen: A Computational Platform Providing Unified Data for Liquid-Crystal Research

Keda Yang[1], Longkun Du[2], Zirui Lv[2], Wangshi Ning[2], Jun Li[2], Qiongyan Shao[1], Jie Qu[1], Lihui Hong[1], Teng Lu[3]*, Xiaoling Xu[1]*, Denghui Li[2]*

[1] Key Laboratory of Artificial Organs and Computational Medicine of Zhejiang Province, Shulan International Medical College, Zhejiang Shuren University, Hangzhou, China.

[2]State Key Laboratory of Chemistry for NBC Hazards Protection, Beijing 102205, China,

[3]Computer Network Information Center, Chinese Academy of Sciences, Beijing 100190, China

* Correspondence: luteng@sccas.cn, ziyao1988@zju.edu.cn, lidenghui@mail.nankai.edu.cn

Supporting Information

Table S1. MesoScreen summary values for compounds 1–36 at 313 K and 1 bar (200 molecules per system). Compound numbers match website oid values (compound 1 = 5CB). Phase is the S plus τ1 heuristic label. Rotational-viscosity entries are apparent 0.5–15 ns fit outputs; [SL] denotes sampling-limited under the volume-specific ΔMSD > 0.02 check; [DG] denotes that the displacement gate is passed but other diagnostics remain unavailable; † marks the 7CB fit as internally inconsistent with its high S. γ1 for isotropic snapshots is not a nematic viscosity.

No.	S	Phase label	V (nm³)	Δα (a.u.)	ΔE (Ha)	Δn	Apparent γ_1 (mPa s)
1	0.519	Nematic	84.45	190.418	0.30989	0.1887	15.5 [DG]
2	0.894	Nematic	96.96	29.747	0.50791	0.0477	1355.5 [SL]

No.	S	Phase label	V (nm^3)	Δα (a.u.)	ΔE (Ha)	Δn	Apparent γ_1 (mPa s)
3	0.112	Weakly nematic	84.36	79.949	0.35758	0.0159	2.0 [DG]
4	0.096	Weakly nematic	77.53	88.320	0.35849	0.0168	2.6 [DG]
5	0.212	Nematic	109.05	97.942	0.34735	0.0300	16.7 [DG]
6	0.052	Isotropic	108.30	121.408	0.34071	0.0096	5.6 [DG]
7	0.859	Nematic	107.21	211.212	0.31057	0.2765	49660.4 [SL]
8	0.068	Isotropic	83.71	183.099	0.30825	0.0243	24.5 [DG]
9	0.072	Isotropic	99.24	185.834	0.31021	0.0221	29.7 [DG]
10	0.096	Weakly nematic	89.18	189.126	0.31277	0.0334	10.1 [DG]
11	0.466	Nematic	90.67	113.904	0.34346	0.0943	105.5 [DG]
12	0.468	Nematic	88.99	192.684	0.31278	0.1641	15.3 [DG]
13	0.612	Nematic	95.05	193.468	0.31006	0.2015	3.1 [DG]†
14	0.892	Nematic	110.62	209.876	0.30983	0.2749	25990.5 [SL]
15	0.580	Nematic	120.60	197.500	0.31374	0.1574	39291.3 [SL]
16	0.800	Nematic	94.19	219.604	0.29564	0.3107	434.9 [SL]
17	0.767	Nematic	97.55	225.806	0.29013	0.2961	515.8 [SL]
18	0.237	Nematic	99.87	223.426	0.29315	0.0897	132.4 [SL]

No.	S	Phase label	V (nm^3)	Δα (a.u.)	ΔE (Ha)	Δn	Apparent γ_1 (mPa s)
19	0.850	Nematic	112.72	207.308	0.31136	0.2550	40017.5 [SL]
20	0.989	Smectic	93.67	453.076	0.27152	0.7821	6454.3 [SL]
21	0.929	Smectic	95.13	493.189	0.26094	0.7916	8374.7 [SL]
22	0.861	Nematic	90.20	361.786	0.26499	0.5575	2497.7 [SL]
23	0.881	Nematic	97.32	485.998	0.25603	0.7186	115.2 [DG]
24	0.805	Nematic	107.39	172.189	0.31217	0.2039	1659.1 [SL]
25	0.470	Nematic	92.33	239.566	0.29523	0.2038	236.5 [SL]
26	0.074	Isotropic	97.28	231.405	0.29279	0.0299	45.3 [DG]
27	0.894	Smectic	126.59	460.767	0.26720	0.5334	3193.6 [SL]
28	0.058	Isotropic	154.98	431.547	0.26840	0.0253	685.5 [SL]
29	0.888	Nematic	126.47	413.004	0.28213	0.4763	48888.4 [SL]
30	0.856	Nematic	137.66	541.620	0.26685	0.5558	32654.5 [SL]
31	0.982	Nematic	130.39	541.521	0.27062	0.6726	19449.0 [SL]
32	0.865	Nematic	125.74	671.183	0.26237	0.7584	18748.0 [SL]
33	0.970	N/Sm crossover	162.74	810.109	0.25976	0.7913	28.7 [DG]
34	0.932	Nematic	142.12	793.390	0.26518	0.8636	141531.6 [SL]

No.	S	Phase label	V (nm^3)	Δα (a.u.)	ΔE (Ha)	Δn	Apparent γ_1 (mPa s)
35	0.869	Nematic	131.73	574.962	0.26713	0.6126	183.4 [SL]
36	0.903	Smectic	90.12	46.963	0.41764	0.0797	261.5 [SL]

The 0.5–15 ns fit interval spans 14.5 ns. Apparent $\gamma 1$ values marked [SL] must not be used for quantitative ranking; [DG] is not equivalent to full validation.

Numbered molecular structures are shown in Figure S1; canonical SMILES, InChIKeys, molecular formulas, molar masses, and mean-volume-derived densities are reported in Table S2. Compounds 8, 9, 10, 1, 12, and 13 are 2CB–7CB. Compound 1 is 5CB. Figures S1 show standardized two-dimensional depictions generated directly from the submitted SMILES. Compound numbers match Tables S1 and S2.

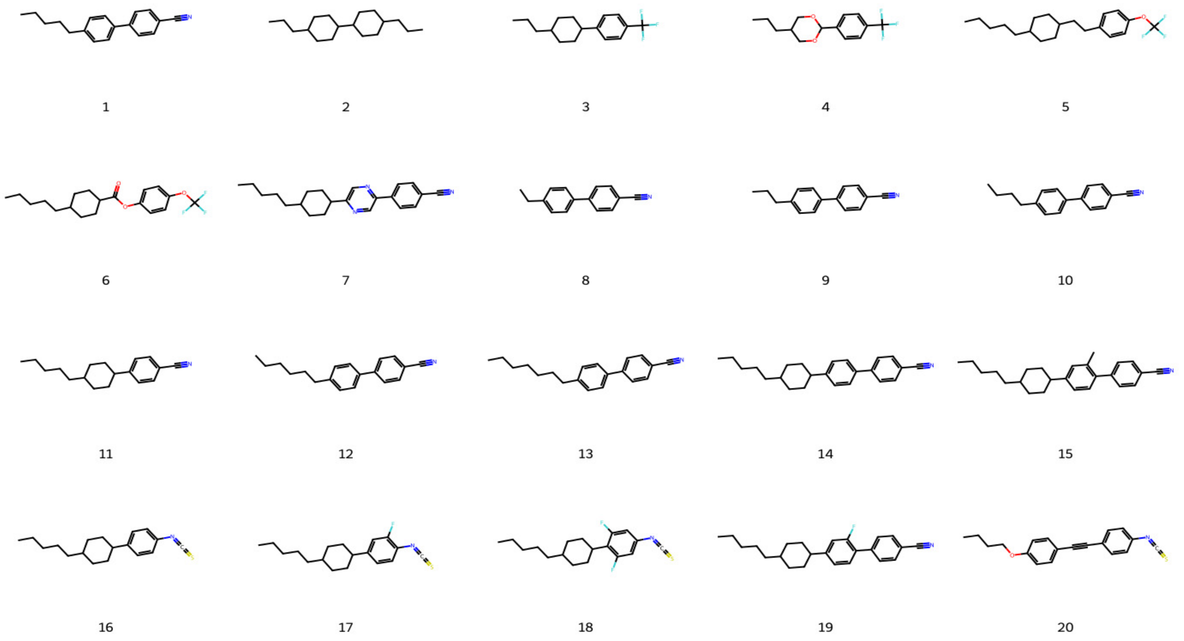

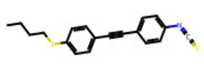

21

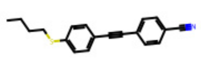

22

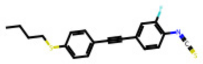

23

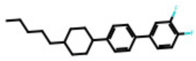

24

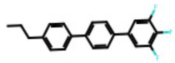

25

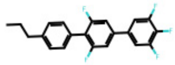

26

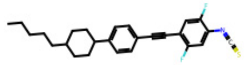

27

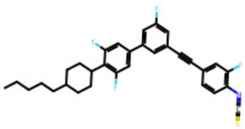

28

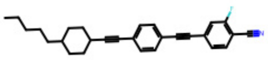

29

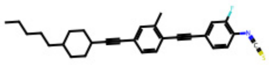

30

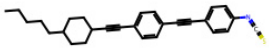

31

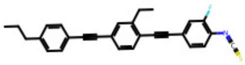

32

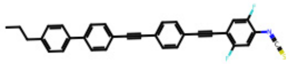

33

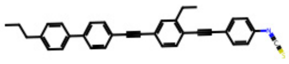

34

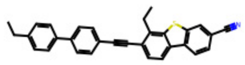

35

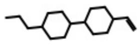

36

Figure S1. Numbered two-dimensional molecular structures for compounds 1–36.

Table S2. Standardized molecular identity and mean-density reconstruction for compounds 1–36. Canonical SMILES and standard InChIKeys were generated from the submitted SMILES strings with RDKit. M is the SMILES-derived molar mass. Mean density was reconstructed as $\rho = 200M/(N_A\langle V\rangle)$ from the Table S1 cell volume; no density uncertainty is implied because volume time series were not supplied.

No.	Formula; M (g mol⁻¹); $\bar{\rho}$ (g cm⁻³)	InChIKey	Canonical SMILES
1	C18H19N; M = 249.357; $\bar{\rho}$ = 0.980	HHPCNRKYVYWYAU-UHFFFAOYSA-N	CCCCCc1ccc(-c2ccc(C#N)cc2)cc1
2	C18H34; M = 250.470; $\bar{\rho}$ = 0.858	QYBFJRPJNNTNOV-UHFFFAOYSA-N	CCCC1CCC(C2CCC(CCC)CC2)CC1
3	C16H21F3; M = 270.338; $\bar{\rho}$ = 1.064	SUGJPZWRDSZSEW-UHFFFAOYSA-N	CCCC1CCC(c2ccc(C(F)(F)F)cc2)CC1

No.	Formula; M (g mol⁻¹); ρ̄ (g cm⁻³)	InChIKey	Canonical SMILES
4	C14H17F3O2; M = 274.282; ρ̄ = 1.175	SZHICIZJAWIIQU-UHFFFAOYSA-N	CCCC1COC(c2ccc(C(F)(F)F)cc2)OC1
5	C20H29F3O; M = 342.445; ρ̄ = 1.043	YVXWCQAAWLFDEC-UHFFFAOYSA-N	CCCCCC1CCC(CCc2ccc(OC(F)(F)F)cc2)CC1
6	C19H25F3O3; M = 358.400; ρ̄ = 1.099	XKWVQFZKPFJBHJ-UHFFFAOYSA-N	CCCCCC1CCC(C(=O)Oc2ccc(OC(F)(F)F)cc2)CC1
7	C22H27N3; M = 333.479; ρ̄ = 1.033	XUYWGIRETOPGCA-UHFFFAOYSA-N	CCCCCC1CCC(c2cnc(-c3ccc(C#N)cc3)cn2)CC1
8	C15H13N; M = 207.276; ρ̄ = 0.822	DLLIPJSMDJCZRF-UHFFFAOYSA-N	CCc1ccc(-c2ccc(C#N)cc2)cc1
9	C16H15N; M = 221.303; ρ̄ = 0.741	XFMPTZWVMVMELB-UHFFFAOYSA-N	CCCc1ccc(-c2ccc(C#N)cc2)cc1
10	C17H17N; M = 235.330; ρ̄ = 0.876	PJPLBHHDTUICNN-UHFFFAOYSA-N	CCCCc1ccc(-c2ccc(C#N)cc2)cc1
11	C18H25N; M = 255.405; ρ̄ = 0.936	FURZYCFZFBYJBT-UHFFFAOYSA-N	CCCCCC1CCC(c2ccc(C#N)cc2)CC1
12	C19H21N; M = 263.384; ρ̄ = 0.983	VADSDVGLFDVIMG-UHFFFAOYSA-N	CCCCCCc1ccc(-c2ccc(C#N)cc2)cc1
13	C20H23N; M = 277.411; ρ̄ = 0.969	ZGOWXOZNUNZPAV-UHFFFAOYSA-N	CCCCCCCc1ccc(-c2ccc(C#N)cc2)cc1
14	C24H29N; M = 331.503; ρ̄ = 0.995	QKEBUASRTJNJJS-UHFFFAOYSA-N	CCCCCC1CCC(c2ccc(-c3ccc(C#N)cc3)cc2)CC1
15	C25H31N; M = 345.530; ρ̄ = 0.952	ZCWVNQYAZUVFJY-UHFFFAOYSA-N	CCCCCC1CCC(c2ccc(-c3ccc(C#N)cc3)c(C)c2)CC1
16	C18H25NS; M = 287.472; ρ̄ = 1.014	SWQXYZYWUZHFGJ-UHFFFAOYSA-N	CCCCCC1CCC(c2ccc(N=C=S)cc2)CC1
17	C18H24FNS; M = 305.462; ρ̄ = 1.040	NMNHWGWAENBRBB-UHFFFAOYSA-N	CCCCCC1CCC(c2ccc(N=C=S)c(F)c2)CC1
18	C18H23F2NS; M = 323.452; ρ̄ = 1.076	JATCHYOGTUPGFA-UHFFFAOYSA-N	CCCCCC1CCC(c2c(F)cc(N=C=S)cc2F)CC1
19	C24H28FN; M = 349.493; ρ̄ = 1.030	FKZBMESICJTAEC-UHFFFAOYSA-N	CCCCCC1CCC(c2ccc(-c3ccc(C#N)cc3)c(F)c2)CC1
20	C19H17NOS; M = 307.418; ρ̄ = 1.090	OMRYAVGAZYUAES-UHFFFAOYSA-N	CCCCOc1ccc(C#Cc2ccc(N=C=S)cc2)cc1
21	C19H17NS2; M = 323.486; ρ̄ = 1.129	FQKWDRPSDRDOFR-UHFFFAOYSA-N	CCCCSc1ccc(C#Cc2ccc(N=C=S)cc2)cc1

No.	Formula; M (g mol^{-1}); $\bar{\rho}$ (g cm^{-3})	InChIKey	Canonical SMILES
22	C19H17NS; M = 291.419; $\bar{\rho}$ = 1.073	HTSWAKOIZJDDLJ-UHFFFAOYSA-N	CCCCSc1ccc(C#Cc2ccc(C#N)cc2)cc1
23	C19H16FNS2; M = 341.476; $\bar{\rho}$ = 1.165	ZKRRTTWUEIHCEW-UHFFFAOYSA-N	CCCCSc1ccc(C#Cc2ccc(N=C=S)c(F)c2)cc1
24	C23H28F2; M = 342.473; $\bar{\rho}$ = 1.059	NQLHGECGZRJQLF-UHFFFAOYSA-N	CCCCCC1CCC(c2ccc(-c3ccc(F)c(F)c3)cc2)CC1
25	C21H17F3; M = 326.361; $\bar{\rho}$ = 1.174	SEZXWSLMESQCCN-UHFFFAOYSA-N	CCCc1ccc(-c2ccc(-c3cc(F)c(F)c(F)c3)cc2)cc1
26	C21H15F5; M = 362.341; $\bar{\rho}$ = 1.237	NNDVDDQJJPMUJR-UHFFFAOYSA-N	CCCc1ccc(-c2c(F)cc(-c3cc(F)c(F)c(F)c3)cc2F)cc1
27	C26H27F2NS; M = 423.572; $\bar{\rho}$ = 1.111	HJYBORIWBVQDEI-UHFFFAOYSA-N	CCCCCC1CCC(c2ccc(C#Cc3cc(F)c(N=C=S)cc3F)cc2)CC1
28	C32H29F4NS; M = 535.650; $\bar{\rho}$ = 1.148	BSKHFLQGGXJJDI-UHFFFAOYSA-N	CCCCCC1CCC(c2c(F)cc(-c3cc(F)cc(C#Cc4ccc(N=C=S)c(F)c4)c3)cc2F)CC1
29	C28H28FN; M = 397.537; $\bar{\rho}$ = 1.044	DXPHCYICAJTURP-UHFFFAOYSA-N	CCCCCC1CCC(C#Cc2ccc(C#Cc3ccc(C#N)c(F)c3)cc2)CC1
30	C29H30FNS; M = 443.631; $\bar{\rho}$ = 1.070	RJEOXJOWWJXPNE-UHFFFAOYSA-N	CCCCCC1CCC(C#Cc2ccc(C#Cc3ccc(N=C=S)c(F)c3)c(C)c2)CC1
31	C28H29NS; M = 411.614; $\bar{\rho}$ = 1.048	JLNOZBHBAYHYTL-UHFFFAOYSA-N	CCCCCC1CCC(C#Cc2ccc(C#Cc3ccc(N=C=S)cc3)cc2)CC1
32	C28H22FNS; M = 423.556; $\bar{\rho}$ = 1.119	NNULVZKQNNOLNL-UHFFFAOYSA-N	CCCc1ccc(C#Cc2ccc(C#Cc3ccc(N=C=S)c(F)c3)c(CC)c2)cc1
33	C32H21F2NS; M = 489.590; $\bar{\rho}$ = 0.999	IBUXMUUGFULAOY-UHFFFAOYSA-N	CCCc1ccc(-c2ccc(C#Cc3ccc(C#Cc4cc(F)c(N=C=S)cc4F)cc3)cc2)cc1
34	C34H27NS; M = 481.664; $\bar{\rho}$ = 1.126	TUSWFUPFPMDSQH-UHFFFAOYSA-N	CCCc1ccc(-c2ccc(C#Cc3ccc(C#Cc4ccc(N=C=S)cc4)c(CC)c3)cc2)cc1

No.	Formula; M (g mol^{-1}); $\bar{\rho}$ (g cm^{-3})	InChIKey	Canonical SMILES
35	$C_{31}H_{23}NS$; M = 441.599; $\bar{\rho}$ = 1.113	WPYACFVJMGFZNR-UHFFFAOYSA-N	CCc1ccc(-<br>c2ccc(C#Cc3ccc4c(sc5cc(C#N)ccc54)c3CC)cc2)cc1
36	$C_{17}H_{30}$; M = 234.427; $\bar{\rho}$ = 0.864	KHDBEDDPFRHGCN-UHFFFAOYSA-N	C=CC1CCC(C2CCC(CCC)CC2)CC1
